# Trends of Prescription Drug Manufacturer Rebates in Commercial Health Insurance Plans, 2015-2019

**DOI:** 10.1001/jamahealthforum.2022.0888

**Published:** 2022-05-06

**Authors:** Elizabeth Plummer, Mariana P. Socal, Jeromie M. Ballreich, Gerard F. Anderson, Ge Bai

**Affiliations:** 1 Neeley School of Business, Texas Christian University, Fort Worth; 2 TCU School of Medicine, Texas Christian University, Fort Worth; 3 Department of Health Policy and Management, Johns Hopkins Bloomberg School of Public Health, Baltimore, Maryland; 4 Johns Hopkins School of Medicine, Baltimore, Maryland; 5 Johns Hopkins Carey Business School, Baltimore, Maryland

## Abstract

This economic evaluation examines the magnitude and trend of prescription drug rebates in commercial markets from 2015 to 2019 and identifies insurance plan factors associated with rebates.

## Introduction

Prescription drug manufacturers routinely offer postsale rebates to pharmacy benefit managers (PBMs) and health insurance plans.^1^ While drug rebates can reduce plans’ net costs, rebates do not reduce patients’ cost sharing, which is usually based on prerebate list prices set by drug companies.^1,2^ Drug rebates can incentivize drug manufacturers to inflate list prices and PBMs to distort drug formularies to favor high list price and high rebate therapies.^1,2,3^ These issues are the focus of recent policy proposals by the US Department of Health and Human Services, as well as federal legislative initiatives.^4^

Recent studies have estimated overall price concessions for prescription drugs by using drug manufacturers’ financial disclosures.^2,3,5^ However, estimated overall price concessions also include coupons, discounts, and other nonrebate items that do not necessarily affect PBMs’ incentives, and it remains unknown what insurance plan characteristics are associated with drug rebates. In this economic evaluation, we examined the magnitude and trend of prescription drug rebates in commercial markets from 2015 to 2019 and identified insurance plan factors associated with rebates.

## Methods

We obtained data from health insurers’ mandatory medical loss ratio (MLR) filings for 2015 through 2019.^6^ Each insurer’s MLR information is provided separately for each state and each market within a state (individual, small group, large group). The present sample includes approximately 2200 unique health plans (70 million covered lives each year) with reported positive prescription drug rebates. Annual prerebate prescription drug spending was approximately $15 billion to $16 billion for individual plans, $13 billion for small group plans, and grew from $33.0 billion to $41.3 billion for large group plans (eMethods 1 and 2 in the Supplement).

We examined median prerebate and postrebate drug cost per covered life (PCL) and median Rebate% (rebate amount divided by prerebate drug cost) over the 5-year period, separately by plan type. We used multivariable linear regression models to examine how Rebate% varied across plan characteristics. We included indicator variables for year, state, and each major insurer. All dollar amounts were adjusted to 2019 values using the Consumer Price Index.

This study follows the Consolidated Health Economic Evaluation Reporting Standards (CHEERS) reporting guidance for economic evaluations. This study does not meet the criteria for human participant research; therefore, institutional review board approval was not sought.

## Results

The Figure shows that, from 2015 to 2019, median prerebate drug cost PCL per year increased by 68.1% (from $734 to $1234) for individual plans, 44.9% (from $752 to $1090) for small group plans, and 23.9% (from $791 to $980) for large group plans, while median postrebate drug cost PCL per year increased by 54.5% (from $644 to $995) for individual plans, 24.0% (from $642 to $796) for small group plans, and only 7.6% (from $686 to $738) for large group plans. Median Rebate% grew steadily for all 3 plan types: from 11.2% to 18.7%, from 13.3% to 22.3%, and from 13.6% to 22.0% for individual plans, small group plans, and large group plans, respectively.

**Figure.**
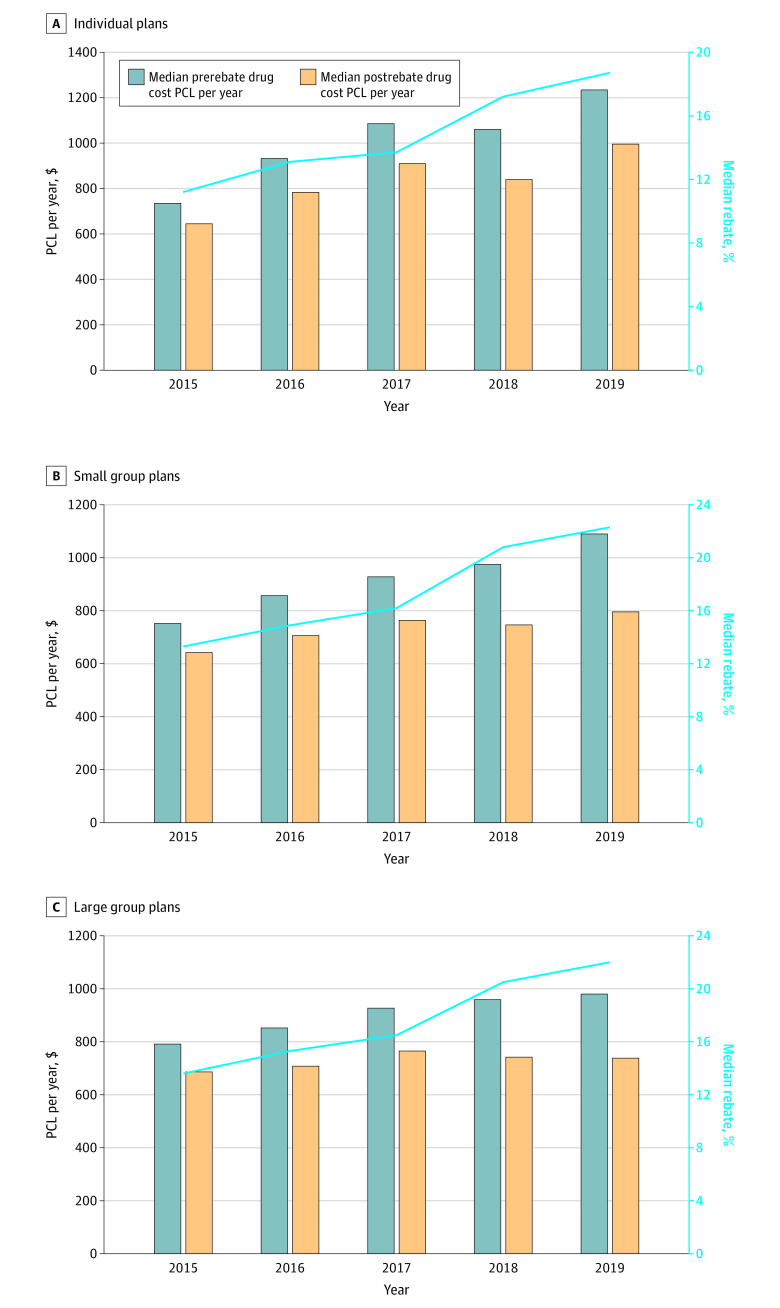
Median Prerebate and Postrebate Drug Cost per Covered Life (PCL) per Year and Median Rebate% for 2015-2019 by Plan Type Rebate% was measured as a plan’s total pharmaceutical rebate divided by its total prerebate prescription drug costs. All dollar amounts were adjusted to 2019 values using the Consumer Price Index. The median covered lives among individual plans for each year between 2015 and 2019 were 3282, 6110, 4955, 4752, and 6318, respectively (A); for small group plans, 4416, 6719, 6017, 6323, and 6085, respectively (B); and for large group plans, 10 760, 13 857, 14 337, 15 098, and 14 020, respectively (C).

Regression results identified drug cost PCL per year as the only plan characteristic statistically significantly associated with Rebate% across all plan types (Table). Holding other factors constant, as drug cost PCL per year grew by 10%, Rebate% dropped by 0.3% for individual plans (*P* < .001), 0.3% for small group plans (*P* < .001), and 0.2% for large group plans (*P* < .001).

**Table.** Results From Regression of Rebate% on Plan-Level Characteristics by Plan Type^a^

Variable	Individual plan		Small group plan		Large group plan	
	Coefficient (95% CI)	*P* value	Coefficient (95% CI)	*P* value	Coefficient (95% CI)	*P* value
Plan characteristics^b^						
Log drug cost per covered life per year	−0.03 (−0.04 to −0.02)	<.001	−0.03 (−0.03 to −0.02)	<.001	−0.02 (−0.02 to −0.01)	<.001
Log No. of covered lives	−0.01 (−0.01 to −0.01)	<.001	−0.00 (−0.00 to −0.00)	<.001	−0.00 (−0.00 to 0.00)	.07
Drug cost to total claims ratio	−0.00 (−0.05 to 0.04)	.82	−0.00 (−0.04 to 0.04)	.99	−0.02 (−0.06 to 0.02)	.22
Major insurer^c^						
Blue Cross Blue Shield	0.05 (0.03 to 0.06)	<.001	0.02 (0.01 to 0.03)	<.001	0.01 (0.01 to 0.02)	<.001
Kaiser Permanente	−0.06 (−0.11 to −0.02)	.008	−0.09 (−0.12 to −0.07)	<.001	−0.09 (−0.11 to −0.07)	<.001
UnitedHealthcare	0.10 (0.09 to 0.12)	<.001	0.14 (0.13 to 0.15)	<.001	0.13 (0.12 to 0.14)	<.001
Health Care Service Corporation	0.05 (−0.01 to 0.10)	.13	0.01 (−0.02 to 0.05)	.40	0.01 (−0.02 to 0.04)	.47
Aetna	0.01 (−0.01 to 0.04)	.24	−0.01 (−0.02 to 0.00)	.13	−0.00 (−0.01 to 0.00)	.50
Humana	0.03 (−0.01 to 0.06)	.11	0.01 (−0.00 to 0.03)	.08	0.02 (0.00 to 0.03)	.006
Cigna	0.02 (−0.02 to 0.05)	.30	0.09 (0.06 to 0.11)	<.001	0.03 (0.02 to 0.04)	<.001
Centene	0.07 (0.04 to 0.11)	<.001	0.01 (−0.02 to 0.05)	.51	−0.02 (−0.04 to 0.00)	.09
Year						
2016	0.02 (0.00 to 0.03)	.03	0.03 (0.02 to 0.03)	<.001	0.02 (0.01 to 0.02)	<.001
2017	0.03 (0.01 to 0.04)	.003	0.03 (0.02 to 0.04)	<.001	0.03 (0.02 to 0.04)	<.001
2018	0.05 (0.03 to 0.07)	<.001	0.06 (0.05 to 0.07)	<.001	0.06 (0.06 to 0.07)	<.001
2019	0.07 (0.06 to 0.09)	<.001	0.07 (0.06 to 0.08)	<.001	0.08 (0.08 to 0.09)	<.001
State fixed effects	Yes	NA	Yes	NA	Yes	NA
*R* ^2^	0.20	NA	0.49	NA	0.52	NA
No. of plan-years	2537	NA	2515	NA	2905	NA

Abbreviation: NA, not applicable.

^a^ Rebate% was measured as a plan’s total pharmaceutical rebate divided by its total prerebate prescription drug costs. All dollar amounts were adjusted to 2019 values using the Consumer Price Index.

^b^ Plan characteristics include a plan’s drug spending (prerebate drug cost per covered life per year), size (No. of covered lives), and the relative importance of prescription-drug spending to all health care spending (ratio of prerebate drug cost to total claims).

^c^ Each of the included insurers had more than 8.5 million covered lives during the sample period across the 3 plan types combined, substantially higher than the approximately 3 million covered lives of the next largest insurer.

## Discussion

Results of this economic evaluation show that from 2015 to 2019, the growth of prerebate prescription drug costs (used for patients’ cost sharing) outpaced the growth of postrebate drug costs for all 3 commercial plan types. The consistently negative association between prerebate drug cost PCL per year and Rebate% documented in this study might reflect the fact that many expensive drugs with little competition rarely offer large manufacturer rebates.^2^ As a limitation of this study, caution is needed in interpreting the results owing to the lack of information from MLR reports on benefit design and drug utilization, especially on the composition of brand-name drugs vs generics. Because rebates are almost uniformly directed at brand-name drugs, the actual magnitude of rebates in commercial plans is higher than Rebate% estimated in this study.

## Notes

Supplement. **eMethods 1.** Data and Sample **eMethods 2.** Regression Model
