# Supplementary material for: Trends of Prescription Drug Manufacturer Rebates in Commercial Health Insurance Plans, 2015-2019
Source: JAMA Health Forum. 2022 May 6;3(5):e220888. doi: 10.1001/jamahealthforum.2022.0888 (PMC9077484; doi:10.1001/jamahealthforum.2022.0888)
Supplement: Supplement. — eMethods 1. Data and Sample eMethods 2. Regression Model [file jamahealthforum-e220888-s001.pdf]

Supplemental Online Content

Plummer E, Socal MP, Ballreich JM, Anderson GF, Bai G. Trends of prescription drug manufacturer rebates in commercial health insurance plans, 2015-2019. *JAMA Health Forum*. 2022;3(5):e220888. doi:10.1001/jamahealthforum.2022.0888

eMethods 1. Data and Sample

eMethods 2. Regression Model

This supplemental material has been provided by the authors to give readers additional information about their work.

© 2022 Plummer E et al. *JAMA Health Forum.*

eMethods 1. Data and Sample

The data used in this study were obtained from health insurers' Medical Loss Ratio (MLR) filings for 2015 through 2019. The Patient Protection and Affordable Care Act (ACA) requires health insurance companies to disclose how much they spend on healthcare and how much they spend on administrative costs. If an insurer spends less than 80% (85% in the large group market) of premiums on qualified medical expenses, the insurer must refund the portion of premiums that exceeded the limit.

To satisfy the MLR disclosure requirements, health insurers must submit annual reports to the Department of Health and Human Services for each market (individual, small group, large group) and state in which an insurer does business. Reporting is on a calendar year basis. Individual plans are issued to individuals, cover the individual and their dependents, and include plans purchased through ACA exchanges and off-exchange. Small group plans are purchased by employers with 50 (100 in some states) or fewer covered employees. Large group plans are purchased by employers with more than 50 (100 in some states) covered employees. All data used in this study was obtained from the MLR filings, including number of covered lives, prescription drug claims (outpatient prescription drugs only), drug manufacturer rebates, and total claims.

We examined all individual, small group, and large group plans that reported positive prescription drug rebates for 2015 through 2019. Our sample includes approximately 2,200 unique health plans across all three plan types, totaling 7,957 plan-years for 2015-2019. Additional summary information on our sample is available upon request.

MLR data can be downloaded from the Public Use Files at http://www.cms.gov/CCIIO/Resources/Data-Resources/mlr.html.

© 2022 Plummer E et al. *JAMA Health Forum.*

eMethods 2. Regression Model

Rebate% is defined as a plan's total rebate amount divided by its pre-rebate drug cost. We used the following model to examine how *Rebate%* varied across plan characteristics:

$$Rebate\% = \text{Log(\$ drug cost per covered life)} + \text{Log (\# of covered lives)} + \text{Drug-cost-to-total-claims ratio} + \text{BCBS} + \text{Kaiser} + \text{UnitedHealth} + \text{HCSC} + \text{Aetna} + \text{Humana} + \text{Cigna} + \text{Centene} + \text{Year}_{2016} + \text{Year}_{2017} + \text{Year}_{2018} + \text{Year}_{2019} + \text{State indicator variables}$$

We estimated the model separately for each market (individual, small group, and large group). All dollar amounts were adjusted to 2019 values using the Consumer Price Index, and all continuous variables were winsorized at the top and bottom 1%.

We include three plan characteristics: Log($ drug cost per covered life) measures a plan's pre-rebate drug spending per covered life; Log(# of covered lives) measures plan size; and drug-cost-to-total-claims ratio measures the relative importance of a plan's pre-rebate prescription drug claims to its overall medical claims. We include an indicator variable for eight insurers that are considered major insurers in this study. Each of these insurers had more than 8.5 million covered lives during our sample period across the three plan types combined, substantially higher than the approximately 3 million covered lives of the next largest insurer. Finally, we include separate indicator variables for years 2016 through 2019 (2015 is the base year), and also indicator variables for each state.

© 2022 Plummer E et al. *JAMA Health Forum.*
